# Does Capillary or Intravenous Collection of Dried Blood Spots Affect the Results of Amino Acid and Acylcarnitine Profile Studied with Tandem Mass Spectrometry?

**DOI:** 10.3390/metabo16040244

**Published:** 2026-04-04

**Authors:** Banu Kadıoğlu Yılmaz, Abdullah Sivrikaya, Ali Ünlü

**Affiliations:** 1 Department of Pediatric Nutrition and Metabolism, Faculty of Medicine, Selçuk University, Konya 42250, Türkiye; 2 Department of Biochemistry, Faculty of Medicine, Selçuk University, Konya 42250, Türkiye; biyokaya@selcuk.edu.tr (A.S.); aunlu@selcuk.edu.tr (A.Ü.)

**Keywords:** acylcarnitine, amino acid, intravenous, capillary, tandem mass spectrometry

## Abstract

**Background and Objectives:** This study investigated whether capillary and intravenous sampling affect acylcarnitine and amino acid profile results analyzed by tandem mass spectrometry. **Methods:** The study included 120 patients either diagnosed with an inherited metabolic disease or undergoing evaluation for a suspected metabolic disorder at the Department of Pediatric Nutrition and Metabolism, Selçuk University Faculty of Medicine. Paired capillary and intravenous blood samples were collected simultaneously, applied to filter paper, and analyzed by LC-MS/MS to determine acylcarnitine and amino acid profiles. **Results:** Significant differences were observed between capillary and intravenous samples for several acylcarnitines, including C0, C2, C8, C8.1, C10, C10.1, C14.1, C16, and C18.1 (*p* < 0.05). In the amino acid profile, arginine, aspartic acid, citrulline, glutamic acid, glycine, leucine + isoleucine, methionine, tyrosine, and the methionine/phenylalanine ratio differed significantly between sampling methods (*p* < 0.05). Despite these differences, Cohen’s kappa analysis showed high agreement between capillary and venous samples for most parameters (78.3–100%) when categorized as low, normal, or high based on reference ranges. Additionally, no significant discrepancies were found in key diagnostic parameters among patients with specific inherited metabolic diseases. **Conclusions:** Although certain acylcarnitine and amino acid levels differed between capillary and intravenous samples, overall diagnostic agreement was high. However, since the study group did not include any patients with fatty acid oxidation disorders, a separate confirmatory study is needed for this condition. Larger multicenter studies involving more patients and a wider range of metabolic disorders are needed to better understand the clinical impact of sampling method on dried blood spot analyses.

## 1. Introduction

Acylcarnitine and amino acid analysis, performed on dried blood samples collected on filter paper using tandem mass spectrometry (MS/MS), is an important test frequently used in the screening and diagnosis of inherited metabolic diseases in pediatric metabolic disease practice [1]. MS/MS analysis studies acylcarnitine profiles and the amounts and ratios of major amino acids [2]. This method can diagnose more than 50 diseases, including aminoacidopathies, organic acidemias, and fatty acid oxidation disorders, which are among the inherited metabolic diseases [1,3]. Therefore, in many European countries and the United States, newborn screening with MS/MS is routinely performed to diagnose inherited metabolic diseases early and initiate treatment [3]. In Türkiye, newborn screening using MS/MS has not yet been incorporated into the national program.

MS/MS analysis is performed with a few drops of capillary blood collected on filter paper [4]. To date, studies in this area have used capillary blood samples, and reference ranges for acylcarnitine and amino acid levels and ratios have been determined based on capillary blood levels [1,2,3,4,5,6].

In the screening and diagnosis of inherited metabolic diseases, clinicians evaluate patients’ capillary metabolic tests and acylcarnitine analysis. They also request numerous tests that require intravenous (IV) access, such as a hemogram, biochemical tests, blood gas analysis, serum amino acid analysis, and measurements of lactate and pyruvate levels, which can contribute to the diagnosis of other diseases [7]. In our hospital, we observe that metabolic tests and acylcarnitine analysis, which require capillary collection, are occasionally obtained via IV access for various reasons, including limited awareness and established practices of frequent intravenous sampling when drawing blood in inpatient units, intensive care units, and outpatient clinics. It is noteworthy that collecting samples on filter paper for MS/MS analysis via this route, while obtaining IV access, also provides convenience for healthcare professionals. Although the field of technology continues to develop new methods to study more metabolites with less blood, a second attempt to collect a capillary blood sample from the same patient creates problems and additional burden on both patients and healthcare personnel because these personnels are more accustomed to the traditional method of collecting blood samples via IV and because examinations for complicated and rare inherited metabolic diseases already require venous blood analysis [7,8,9].

MS/MS analyses were performed on capillary blood samples, and reference values in the guidelines were derived from capillary blood levels [1,2]. Studies in the literature have compared biochemical and metabolic analyses of blood samples collected by capillary and intravenous methods. Still, there are no guidelines for the intravenous route in MS/MS analyses, nor are there reference values for acylcarnitine and amino acid levels obtained intravenously [8,10,11,12,13]. In a study evaluating 54 patient samples diagnosed with inherited metabolic diseases, acylcarnitine concentrations in dried blood samples were compared with those in plasma using HPLC/MS/MS; however, no comparisons were made for amino acid parameters [13]. In another study, the results of Ultra-High-Pressure Liquid Chromatography (UHPLC) were compared between Touch-Activated Phlebotomy (TAP) and intravenous blood samples from only five healthy volunteers [8]. This study did not use routine LC/MS/MS analysis; it was conducted with only five healthy volunteers, excluding patients who might have pathological findings [8]. Studies in this area have not yet addressed the lack of information in the literature on the interpretation of LC/MS/MS results from intravenous samples.

In our study, we measured amino acid and acylcarnitine levels in blood samples collected simultaneously from patients via capillary and intravenous routes during routine blood collection using a tandem mass spectrometer and compared the results. We investigated whether there is a difference between capillary and intravenous sampling.

## 2. Materials and Methods

### 2.1. Study Group

This prospective cross-sectional study was conducted between 30 November 2024 and 30 October 2025. The study was carried out after approval from the Selçuk University Faculty of Medicine local ethics committee (Date: 10 September 2024, Decision No: 2024/442) and the Selçuk University Scientific Research Foundation (BAP Project No: 24401236). Informed consent was obtained from all subjects and from their legal caregivers involved in the study.

Patients aged 0–216 months who were followed up at the Pediatric Nutrition and Metabolism Department of Selçuk University Faculty of Medicine Hospital with suspected or confirmed inherited metabolic diseases and who applied for follow-up were included in the study. This group of patients included those with a definite diagnosis of hereditary metabolic diseases based on biochemical and genetic evidence (phenylketonuria (PKU), hyperphenylalaninemia (HPA), biotinidase deficiency, glutaric aciduria type 1, methylmalonic acidemia (MMA), isovaleric acidemia, urea cycle disorders, homocystinuria, mitochondrial diseases, etc.) and those who presented for further investigation and testing for various reasons related to possible inherited metabolic diseases (seizures, elevated transaminases, malnutrition, vomiting, jaundice, etc.).

Patients aged 18 years or older, those who did not sign the consent form, and those whose samples were unsuitable for analysis were excluded from the study. The patients included in the study were divided into four age groups (≥0–<1 month, ≥1–<24 months, ≥24–<60 months, ≥60–<216 months), with an equal number of males and females in each group.

### 2.2. Sample Collection

Samples were collected simultaneously from study patients via both capillary and intravenous routes onto filter paper. In the patients included in the study, after blood was drawn from the fingertip or heel using the capillary method during routine check-ups or further investigations, dried blood spots were collected as described in the literature (by placing one drop on each circle on the filter paper, filling at least two circular rings on the filter paper and penetrating to the other side of the paper) [14,15].

Simultaneously, while intravenous blood was collected for other biochemical and metabolic tests, a dried blood spot was obtained from the EDTA tube by pipetting the sample onto filter paper. In the literature, it has been mentioned that some endocannabinoids, fatty acid oxidation, and urea cycle submetabolites may exhibit EDTA-induced changes in a study using an EDTA-containing sample [16]. In another study investigating the direct effect of EDTA on newborn screening using tandem mass spectrometry with DBS, the authors showed that parameters such as biotinidase activity, 17-hydroxyprogesterone, TSH, and porphobilinogen synthase activity were affected, but they found that amino acid and acylcarnitine quantification were not affected by EDTA [17]. In addition, the American College of Medical Genetics and Genomics (ACMG) 2020 update also states that the use of EDTA-containing plasma samples in acylcarnitine analysis is “acceptable” [18]. The venous and capillary samples collected simultaneously on filter paper were sent to the Biochemistry Department Laboratory of Selçuk University Faculty of Medicine for analysis of acylcarnitine and amino acid levels using Tandem Mass Spectrometry. In our study, blood samples were applied to DBS cards and allowed to dry completely at room temperature, horizontally, and without exposure to heat or light for at least 3 h. After drying, the samples were placed in zip-lock bags and stored at +4 °C until analysis.

### 2.3. Sample Analysis

Samples collected simultaneously from patients via capillary and venous routes onto filter paper were analyzed at the Medical Biochemistry Laboratory of Selçuk University Faculty of Medicine. LC-MS/MS analysis parameters included: acylcarnitine profiles (C0 Free, C2 Acetyl, C3 Propionyl, C4 Butyrl, C5:1, C5 Isovaleryl, C5 DC Glutaryl, C5 OH 3OH Isovaleryl, C6 Hexanoyl, C8 Octanoly, C8:1 Octenoyl, C10 Decanoyl, C10:1 Decanoyl, C12 Dodecayl, C14 Myristoyl, C14:1 Myristoyl, C14:2 Myristoyl, C16 Palmitoleyl, C16:1 Palmitoleyl, C18:2 Linoleyl, C18 Stearoyl, C18:1 Oleyl carnitine) and the amount of amino acids and their ratios to each other (Aspartic Acid (Asp), Arginine (Arg), Alanine (Ala), Citrulline (Cit), Glutamic Acid (Glu), Glycine (Gly), Leucine (Leu) + Isoleucine (Ileu), Methionine (Met), Phenylalanine (Phe), Tyrosine (Tyr), Valine (Val), Phe\Tyr, Leu\Phe, Met\Phe were studied. Hematocrit (Hct) and hemoglobin (Hb) levels were also measured simultaneously in the patients.

Measurements of samples collected via both capillary and venous routes were performed on filter paper using the AB-Sciex API 3200 LC-MS/MS instrument located in the metabolism laboratory of the Department of Biochemistry, Selçuk University Faculty of Medicine. A linearity study of the method, which is already in routine use in our biochemistry laboratory using the AB-Sciex API 3200 LC-MS/MS instrument, was conducted to verify the method.

The pre-processing steps for acylcarnitine and amino acid analyses were performed as follows: Dried blood was punched into plate wells using a puncher; 200 μL of standard mix solution was added to each well. The plates were sealed with their own caps and placed in a shaker (37 °C, 26 min). The solvent standard was put into the empty wells. The plate was placed in an evaporation apparatus and evaporated under nitrogen gas. After evaporation, 60 μL of derivative solution was added. The plates were sealed and placed in an oven (65 °C, 15 min). Subsequently, evaporation was repeated under nitrogen; 100 μL of solvent solution (acetonitrile/water) was added. The plate was gently mixed for 20 s, placed in an autosampler, and injected into the instrument.

Intravenous and capillary acylcarnitine and amino acid parameters, along with patients’ definitive diagnoses (if any), reasons for referral, age, gender, and other sociodemographic information, were recorded from the patient’s file.

### 2.4. Statistical Analysis

Statistical analysis of the data was performed using SPSS 26.0 (IBM Corp., Armonk, NY, USA). Descriptive statistics were presented as mean ± standard deviation and median (minimum–maximum) values. Paired *t*-tests were used to compare venous and capillary measurements across continuous variables, or the Wilcoxon signed-ranked test was applied when the distribution was not normal. The kappa coefficient was calculated to assess agreement among the low-, normal-, and high-coded classifications of the parameters, and the corresponding percentages of agreement were reported. Furthermore, *p*-values were reported based on the chi-square test for statistical significance of the agreement. Box-plot graphs were created for parameters where significant differences were found in the comparison of venous and capillary measurements; venous values were shown in black, and capillary values in red. In addition, the relationships between venous and capillary parameters were examined using Pearson correlation analysis, and the results were visualized with heat maps. A *p*-value < 0.05 was considered significant in all statistical tests.

## 3. Results

### 3.1. Assessment of the Age, Gender, and Diagnoses of the Study Group

The study included 120 patients aged 0–216 months, 60 female and 60 male. Among all patients included in the study, no statistically significant difference in age was observed between females and males (*p* = 0.688). In the analysis of subgroups by age, the mean age of females was significantly higher than that of males in the ≥1–<24 months (*p* = 0.039) and ≥24–<60 months (*p* = 0.034) age groups. Demographic information for patients, divided into age subgroups, is summarized in Table 1.

**Figure 1:**
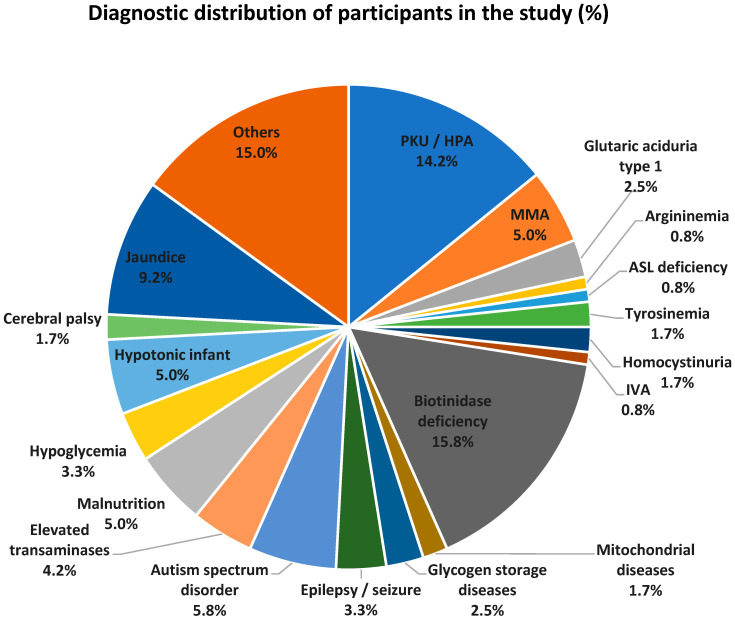
The percentages of definitive diagnoses and reasons for referral among patients included in the study are summarized. IVA: isovaleric acidemia, MMA: methylmalonic acidemia, PKU: phenylketonuria, HPA: hyperphenylalaninemia, ASL: argininosuccinate lyase.

The percentage distribution of reasons for referral (based on the main symptoms and findings or definitive diagnoses) among patients included in the study is summarized in Figure 1. Among patients with inherited metabolic diseases, the most frequent diagnoses were biotinidase deficiency (15.8%), PKU + HPA (14.2%), and MMA (5%). Seventeen patients were followed up with a diagnosis of PKU + HPA; 19 patients with partial or severe biotinidase deficiency; and 6 patients with MMA (3 methylmalonic acidemia, 1 Cobalamin-A defect, 1 Cobalamin-B defect, 1 methylmalonic coA epimerase deficiency). In the groups without a definitive diagnosis of hereditary metabolic disease, the most frequent reasons for referral were jaundice (11 patients), autism spectrum disorder (7 patients), malnutrition (6 patients), and hypotonic infant (6 patients). Other reasons for referral included vomiting, hydrops fetalis, perinatal asphyxia, neonatal sepsis, anorexia, and speech delay.

### 3.2. Comparison of Acylcarnitine and Amino Acid Analyses Obtained by Venous and Capillary Methods in the Study Group

**Figure 2:**
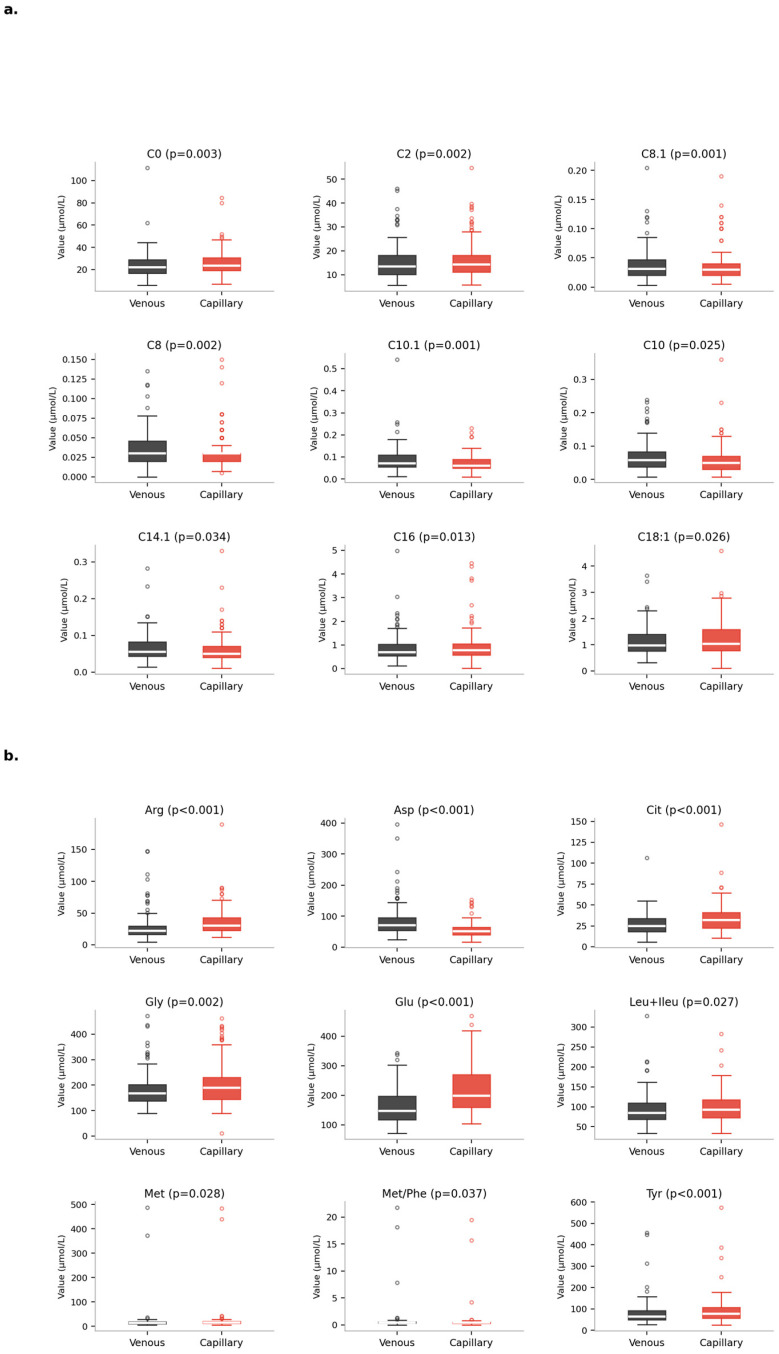
Graphical presentation of statistically significant parameters in acylcarnitine and amino acid parameters studied using venous and capillary methods: (**a**) acylcarnitine parameters, (**b**) amino acid parameters.

Tandem mass spectrometry was used to analyze venous and capillary acylcarnitine and amino acid parameters in 120 patients, who were sampled simultaneously. The mean ± SD, median, minimum, and maximum values, and *p*-values of each parameter are summarized in Table 2. Statistically significant differences were found between the levels of C0, C2, C8, C8.1, C10, C10.1, C14.1, C16, and C18.1 in the acylcarnitine profile of dry blood samples obtained intravenously and capillary (*p* < 0.05). Of these, C0, C2, C16, and C18.1 acylcarnitine parameters were found to be lower in dry blood samples obtained intravenously, while C8, C8.1, C10, C10.1, and C14.1 acylcarnitine parameters were found to be lower in dry blood samples obtained via capillary access. No statistically significant difference was found between dry blood samples obtained via intravenous and capillary methods for other acylcarnitine parameters. In the amino acid profile, statistically significant differences were found in the levels of arginine, aspartic acid, citrulline, glutamic acid, glycine, leucine + isoleucine, methionine, tyrosine, and methionine/phenylalanine in dry blood samples obtained via venous and capillary access (*p* < 0.05). While lower levels of arginine, citrulline, glutamic acid, glycine, leucine + isoleucine, methionine, and tyrosine were detected in dry blood samples obtained intravenously, aspartic acid and the methionine/phenylalanine ratio were statistically significantly lower in dry blood samples obtained via capillary administration. A graphical representation of the statistically significant differences in acylcarnitine and amino acid parameters measured by intravenous and capillary methods is shown in Figure 2.

Patients’ hematocrit (Hct) and hemoglobin (Hb) levels were also measured simultaneously. For Hct (%) the mean ± SD was 39.54 ± 6.64, median 37.9, min. 25, and max. 59.5; for Hb (g/dL), the mean ± SD was 13.21 ± 2.32, median 12.6, min. 8.6, and max. 20.4. The relationship between Htc and Δ was investigated using Spearman correlation and simple linear regression by creating a variable Δ = Capillary − Venous difference for each metabolite. Of the 37 parameters examined, only 3 (8.1%) showed a statistically significant Spearman correlation (ρ) between Htc and Δ: C4 (ρ = 0.184; *p* = 0.045), C6 (ρ = −0.211; *p* = 0.021), and C18:1 (ρ = 0.200; *p* = 0.028). However, the effect size was very low in all of these correlations (R^2^ < 0.05); Htc explains at most 4.2% of the variance in the venous–capillary difference. Our study showed that hematocrit levels did not systematically affect differences in venous–capillary measurements.

One hundred twenty patients were evaluated according to their diagnoses and presenting symptoms. Those with a definitive diagnosis of inherited metabolic disease (based on genetic analysis and metabolic tests) were divided into two groups: those expected to show pathological findings on tandem mass spectrometry and those without a suspected inherited metabolic disease and without such findings. These two groups were then evaluated with respect to venous and capillary acylcarnitine and amino acid parameters. In tandem mass acylcarnitine and amino acid analyses of patients followed up with a definitive diagnosis of inherited metabolic disease (n = 33; PKU + HPA:17, MMA:6, glutaric aciduria type 1: 3, argininemia:1, argininosuccinate lyase deficiency:1, hereditary tyrosinemia:2, homocystinuria:2, isovaleric acidemia:1), no statistically significant difference was found in the measurements of C0, C2, C3, C4, C5.1, C5, C5DC, C5OH, C6, C14.2, C16, C16.1, C18:2, C18, C18:1, alanine, glycine, leucine + isoleucine, methionine, phenylalanine, tyrosine, valine, phe/tyr, and ASA obtained via venous and capillary methods (*p* > 0.05). In contrast, significant differences were found in C8, C8.1, C10, C10.1, C12, C14, C14.1, arginine, aspartic acid, citrulline, glutamic acid, leu/phe, and met/phe values (*p* < 0.05). Among these parameters, arginine, citrulline, and glutamic acid were found to be higher in capillary measurements, while C8, C8.1, C10, C10.1, C12, C14, C14.1, aspartic acid, leu/phe, and met/phe were found to be lower in capillaries compared to venous measurements.

When dry blood samples obtained intravenously and capillary from patients diagnosed with hereditary metabolic diseases were examined, no statistically significant differences were found between the groups in the phe amino acid levels (*p* = 0.431) and phe/tyr ratio (*p* = 0.517) of 17 patients diagnosed with PKU and HPA, in the C3 acylcarnitine levels of 6 patients diagnosed with MMA (*p* = 0.688), in the C5-DC acylcarnitine levels of 3 patients diagnosed with glutaric aciduria type 1 (*p* = 0.374), in the tyrosine amino acid levels of 2 patients followed with hereditary tyrosinemia type 1 (*p* = 0.186), and in the methionine amino acid levels of 2 patients followed with homocystinuria (*p* = 0.655). Because there was one patient each with argininemia, argininosuccinate lyase deficiency, and isovaleric acidemia, *p*-values for this group could not be calculated. The levels and *p*-values of disease-specific parameters by diagnosis of specific inherited metabolic disease are summarized in Table 3.

When the 120 patients in the study group were further divided into 4 subgroups according to their age (≥0–<1 month, ≥1–<24 months, ≥24–<60 months, ≥60–<216 months), with 15 females and 15 males in each group, the parameters that showed statistically significant differences and their *p*-values are summarized in Table 4.

Consistency analysis was performed for each age subgroup (n = 30) using the Wilcoxon test and the median Δ direction of all metabolites for parameters that were higher or lower in capillary versus venous samples, according to the age subgroups shown in Table 4. In the consistency analysis, directional inconsistency was detected between age subgroups in 8 parameters (C2, C3, C8, C8.1, C12, C14, C16, and C18). The consistency analysis of the parameters in age subgroups is presented in Table 5.

### 3.3. Evaluation of Acylcarnitine and Amino Acid Profiles Obtained via Venous and Capillary Methods by the Study Group Using Cohen’s Kappa Analysis

According to the reference ranges of venous and capillary parameters of the 120 patients included in the study, the agreement between parameter values classified as within the reference range (normal), below the reference range, or above the reference range was evaluated using Cohen’s kappa analysis.

High-level and statistically significant kappa values indicating strong agreement were obtained for the parameters C0 (κ = 0.653, 96.7% agreement; *p* < 0.001), C3 (κ = 0.678, 92.5% agreement; *p* < 0.001), C6 (κ = 0.797, 99.2% agreement; *p* < 0.001), and Phe/Tyr (κ = 0.658, 92.5% agreement; *p* < 0.001). In addition, 100% agreement was observed for the measurements of C5DC, C5OH, C8.1, C10, C12, C14.1, C18, and Met/Phe, and the kappa coefficient for these parameters was calculated as 1.00.

Parameters showing moderate agreement were C5 (κ = 0.494, 98.3% agreement; *p* = 0.164), C16 (κ = 0.555, 86.7% agreement; *p* < 0.001), C18:2 (κ = 0.477, 78.3% agreement; *p* < 0.001), Leucine + Isoleucine (κ = 0.492, 98.3% agreement; *p* = 0.168), and Valine (κ = 0.500, 79.2% agreement; *p* < 0.001). Although some of these parameters showed high percentage agreement, no statistically significant difference was detected.

Parameters with lower levels of agreement were Aspartic acid (κ = 0.224, 85% agreement; *p* = 0.184), Alanine (κ = 0.343, 78.3% agreement; *p* = 0.003), Glycine (κ = 0.335, 94.2% agreement; *p* = 0.169), and ASA (κ = 0.318, 96.7% agreement; *p* = 0.342). In addition, the kappa coefficient was calculated as 0 for VC4, VC5.1, VC8, VC10.1, VC14, VC14.2, and VC16.1, and as −0.01 for Arginine. The fact that the Kappa coefficient is close to zero for these parameters indicates that no better-than-chance fit could be demonstrated. This is due to the very low number of cases with out-of-reference values for these metabolites in the study group. Although the percentage agreement between venous and capillary measurements was high for these parameters, the kappa values were not statistically significant due to class-distribution imbalances. Overall, a high level of agreement was observed between venous and capillary measurements for most parameters, and the homogeneity of distributions across the low/normal/high categories influenced the statistical significance of the kappa coefficients.

### 3.4. Evaluation of Acylcarnitine and Amino Acid Parameters Obtained by Venous and Capillary Methods Using Correlation Analysis

**Figure 3:**
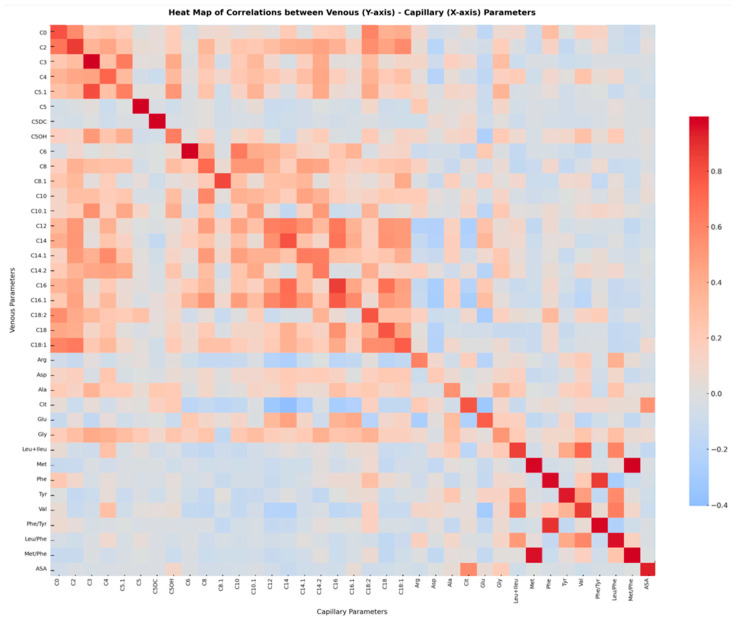
In the figure, acylcarnitine and amino acid parameters obtained via the venous route are shown on the *y*-axis, while parameters obtained via the capillary route are shown on the *x*-axis. Values with high correlation coefficients close to 1 are displayed in red on the heatmap, whereas values with low correlation coefficients are shown in blue. Arg; arginine, Asp; aspartic acid, Ala; alanine, Cit; citrulline, Glu; glutamic acid, Gly; glycine, Leu + Ileu; leucine + isoleucine, Met; methionine, Phe; phenylalanine, Tyr; tyrosine, Val; valine, ASA; argininosuccinic acid.

When the correlation coefficients of venous and capillary measurements were examined, the highest agreement was observed for the C18 (r = 1.00) and Met/Phe (r ≈ 1.00) parameters, while the lowest correlations were found for C18:2 (r ≈ 0.48) and Leucine + Isoleucine (r ≈ 0.49). Overall, the correlation was positive and high across most parameters. A heatmap showing correlations between acylcarnitine and amino acid parameter values in venous and capillary dried blood samples is presented in Figure 3.

## 4. Discussion

In this study, we compared acylcarnitine and amino acid profiles in dried blood samples obtained via capillary and intravenous methods. We evaluated inter-method agreement analyses and correlations of acylcarnitine and amino acid profile parameters in both patients diagnosed with inherited metabolic diseases and those without such diagnoses. In this way, we assessed the usability of dried blood samples obtained via intravenous sampling for the diagnosis of inherited metabolic diseases.

In our study, differences were identified between results obtained from dried blood samples collected intravenously and from capillary blood for certain parameters in the acylcarnitine profile. We found that the C0, C2, C16, and C18.1 parameters were lower in dried blood samples obtained intravenously, whereas the C8, C8.1, C10, C10.1, and C14.1 parameters were lower in dried blood samples obtained by capillary. In a study conducted by de Sain-van der Velden MG et al., comparing acylcarnitine parameters in plasma and dried blood samples analyzed using the HPLC method, similar to our findings, C2, C16, and C18:1 parameters were reported to be lower in plasma samples, while C8, C8.1, C10.1, and C14.1 parameters were lower in dried blood samples [13]. Unlike in our study, C10 values were reported to be similar in plasma and capillary samples, and free carnitine (C0) concentrations were 36% higher in plasma samples; however, in patients with CPT-1 deficiency, free carnitine levels were higher in dried blood samples [13]. In that study, the inherited metabolic disease group consisted of patients with fatty acid oxidation defects (CPT-1 deficiency, CPT-2 deficiency, VLCAD deficiency, long-chain 3-hydroxyacyl-CoA dehydrogenase [LCHAD] deficiency, and medium-chain acyl-CoA dehydrogenase [MCAD] deficiency) and organic acidemias (propionic acidemia, methylmalonic acidemia [MMA], glutaric acidemia type I [GA-I], and β-ketothiolase [βKT] deficiency) [13]. In contrast, the inherited metabolic disease group in our study comprised patients with PKU + HPA, MMA, glutaric aciduria type I, hereditary tyrosinemia type I, homocystinuria, argininemia, argininosuccinate lyase deficiency, and isovaleric acidemia; none had a fatty acid oxidation defect. The differences in free carnitine levels between our study and that of de Sain-van der Velden et al. may therefore be attributed to differences in disease composition and the absence of fatty acid oxidation defects in our cohort. As in the study by de Sain-van der Velden et al., we believe that evaluating patients with and without inherited metabolic diseases together in our study contributed to the similarity of other acylcarnitine profile results and supports the existing literature.

In our study, differences were also observed between dried blood samples obtained intravenously and from capillaries for certain parameters in the amino acid profiles. We found that arginine, citrulline, glutamic acid, glycine, leucine + isoleucine, methionine, and tyrosine levels were lower in intravenously obtained dried blood samples, whereas aspartic acid and the methionine/phenylalanine ratio were lower in capillary dried blood samples. In a study conducted by Catala A et al., concentrations of 45 metabolites within acylcarnitine and amino acid profiles were compared using ultra-high-performance liquid chromatography (UHPLC) in dried blood samples obtained from five healthy volunteers using conventional and TAP methods [8]. In that study, variability was observed in valine (TAP/conventional blood collection fold change = 0.78; *p* = 0.01), unlike our findings, and in arginine (TAP/conventional blood collection fold change = 1.49; *p* = 0.02), similar to our results [8]. Additionally, correlation analyses of TAP and conventional blood collection methods showed the highest correlations for carnitine (r = 0.984) and taurodeoxycholate (r = 0.991), and the lowest correlations for palmitate, histidine, and aspartic acid [8]. In contrast, in our study, the highest correlations between intravenous and capillary measurements were observed for C18 (r = 1.00) and Met/Phe (r ≈ 1.00), while the lowest correlations were found for C18:2 (r ≈ 0.48) and leucine + isoleucine (r ≈ 0.49). Although capillary and intravenous dried blood samples were collected simultaneously from patients in our study, the time intervals before laboratory analysis varied. In our study, the analysis time interval varied between 4 and 21 days.

The literature reports that glutamic acid levels may be elevated due to prolonged sample storage at room temperature, hemolysis, and platelet contamination [19,20,21]. It has also been reported that arginine levels may decrease, particularly due to prolonged storage at room temperature [21]. These factors may explain the differences observed in glutamic acid, arginine, and other amino acid levels between capillary and intravenous dried blood samples in our study. Furthermore, excessive squeezing of the heel or fingertip during capillary blood collection and prolonged sampling time may increase aspartic acid levels [22]. In our patient group, capillary dried blood samples were collected rapidly by experienced personnel, which may explain the lower aspartic acid levels observed in these samples.

In a study evaluating proteomic-type metabolomic measurements using capillary and venous methods, similar to our findings, high correlations between capillary and venous measurements were reported; however, proteomic values were higher in capillary measurements [23]. Capillary blood samples contain a mixture of blood from arterioles, venules, and capillaries, whereas venous samples contain blood that has been utilized by tissues and returned to circulation [24,25]. Since amino acids are protein-based molecules and are consumed by tissues, lower levels in intravenous samples and higher levels in capillary samples may be expected, as observed in our study.

In a study conducted on capillary samples to establish age- and sex-specific reference ranges for amino acids and acylcarnitines using tandem MS, more pronounced age-specific variations were reported for amino acids, with most amino acids (glycine, leucine + isoleucine, ornithine, and tyrosine) showing higher concentrations in infants ≤1 month of age, and arginine levels being higher in individuals aged 11–18 years compared with infants [5]. In the same study, acylcarnitine profile results showed that C0, C5-OH, C10, and C10:1 concentrations were lower in infants and increased with age, while C2, C3, C5, C6, C12, C14, C16, C16:1, C18, and C18:1 concentrations decreased with age [5]. Similarly, in our study, we identified potential differences in acylcarnitine and amino acid profiles between intravenous and capillary samples across age subgroups (≥0–<1 month, ≥1–<24 months, ≥24–<60 months, ≥60–<216 months). Due to the lack of reference ranges for venous dried blood samples in the literature, capillary dried blood sample reference values were used in our analysis. The variation in high and low acylcarnitine and amino acid profile parameters across age subgroups in both capillary and intravenous dried blood samples appears to be attributable to age- and sex-related variability in reference ranges, as reported in the literature.

In our study, when parameter analyses were examined in terms of consistency according to age subgroups, it was observed that most of the parameters showing consistent direction in all age groups were also statistically significant in every age group. Arg, Cit, Glu, Tyr, and Phe were always found to be capillary > venous; Asp and Leu/Phe ratio were always found to be venous > capillary. This consistency reliably demonstrates that true biological differences exist in these metabolites independently of age. Consequently, the explanation for the directional inconsistencies in our study is that small subgroup sizes (n = 30) lead to insufficient statistical power. For metabolites with large effect sizes, the direction is consistent and significant across all age groups; for those with small effect sizes, random fluctuations dominate. In our study, inconsistencies were detected in the acylcarnitine parameters in age subgroup analyses; while capillary > venous results were mostly observed, venous > capillary results were particularly seen in the 60–216 month age range. In a study involving 163 healthy individuals examining plasma acylcarnitines, it was found that long-chain acylcarnitines increased with age in healthy subjects, and most odd-chain acylcarnitines decreased [26]. It has been suggested that mitochondrial dysfunction or mitochondrial reprogramming associated with aging may be the cause of this [26]. In a study investigating capillary-venous differences in 10 healthy male patients using lipidomics, researchers suggested that lipidomics, including acylcarnitines, exhibited similar results in capillary and venous sampling, and that some statistically significant differences observed in acylcarnitines might be due to random variations [12]. In the literature, the changes in acylcarnitine levels across age, disease, and mitochondrial responses to environmental factors remain poorly explained.

de Sain-van der Velden MG et al. reported that free carnitine concentrations in dried blood samples were fourfold higher than plasma concentrations in patients with carnitine palmitoyltransferase 1 (CPT-1) deficiency, and that primary acylcarnitine diagnostic markers were abnormal in plasma but normal in dried blood samples in CPT-2 deficiency [13]. Based on these findings, the authors emphasized that CPT-1 deficiency might be missed when evaluating acylcarnitine markers using plasma samples, while CPT-2 deficiency might be overlooked when using dried blood samples [13]. In contrast, in our study, disease-specific diagnostic parameters were found to be similar between capillary and intravenous samples: phenylalanine levels (*p* = 0.431) and Phe/Tyr ratios (*p* = 0.517) in patients with PKU + HPA, C3 acylcarnitine levels (*p* = 0.688) in patients with MMA, C5-DC acylcarnitine levels (*p* = 0.374) in patients with glutaric aciduria type I, tyrosine levels (*p* = 0.186) in patients with hereditary tyrosinemia type I, and methionine levels (*p* = 0.655) in patients with homocystinuria. Additionally, we found that capillary and intravenous dried blood samples showed generally high agreement (78.3–100%) and high correlation when classified as high, low, or normal according to reference ranges. Based on these findings, we conclude that dried blood samples obtained via the intravenous method can be used in the diagnosis of inherited metabolic diseases and yield results comparable to those obtained via the capillary method.

In our study although a very high correlation was observed for some parameters such as C18, Phe, Tyr, the Bland–Altman analysis revealed a bias and quite wide limits of agreement. This wide limits of agreement indicates that despite high correlation, significant measurement deviations can occur at the individual level, emphasizing the need for caution in clinical decision-making, especially with metabolites that have narrow reference ranges.

### 4.1. Suggestions for Future Research

Future studies evaluating dried blood samples obtained via the intravenous route using LC/MS/MS should focus on establishing reference ranges for intravenously obtained dried blood samples. If our findings are supported by multicenter studies including larger populations with a broader spectrum of inherited metabolic diseases, the use of intravenously obtained dried blood samples for screening and diagnosis of inherited metabolic diseases may be considered.

### 4.2. Limitations

Our study had several limitations. One limitation was the absence of patients with fatty acid oxidation defects, which could potentially influence acylcarnitine profile results. Another limitation was the inclusion of only one patient each with argininemia, argininosuccinate lyase deficiency, and isovaleric acidemia, which precluded statistical analysis of these subgroups. Additionally, due to the non-homogeneous distribution of samples with out-of-range values, kappa analyses could not be performed for certain acylcarnitine and amino acid parameters (C2, C5-OH, C8.1, C10, and C14.1). The lack of reported reference ranges for intravenously obtained dried blood samples in the literature, necessitating the use of capillary dried blood sample reference ranges, represents another limitation of our study. This situation carries the clinical risk of misclassification when using the capillary-derived cutoff point for venous samples. For this reason, as we emphasized in the suggestions for future research section, there is a need for studies that focus on reference ranges for dried blood samples obtained via intravenous sampling. The fact that our study included only one patient each from the argininemia, ASL deficiency, and IVA diagnostic groups means that our findings for these disease groups are at the “preliminary observations” level rather than diagnostic.

## 5. Conclusions

In our study, we found that certain acylcarnitine and amino acid profile parameters differed between capillary and intravenous dried blood samples. Despite these differences, the generally high agreement and high correlation between parameters in capillary and intravenous dried blood samples, as well as the similarity of disease-specific diagnostic parameters between methods, suggest that dried blood samples obtained via the intravenous route can be used for screening and diagnosis of inherited metabolic diseases. However, some acylcarnitine parameters used to diagnose fatty acid oxidation disorders showed lower agreement, and the mean values were statistically significantly different between capillary and venous samples in our study. Since no patients with fatty acid oxidation disorders were included in our study group, the use of venous rather than capillary samples for diagnosing these disorders cannot yet be considered reliable. Although our study provides information on amino acid and acylcarnitine profiles in specific aminoacidopathies and organic acidemias, further studies, including patients with fatty acid oxidation disorders, are needed, as this group was not represented in our cohort. Multicenter studies with larger patient populations and a wider range of inherited metabolic diseases are needed to further validate these findings.

## Figures and Tables

**Table 1:** Distribution of 120 patients according to gender and age.

*Age (Months)*	Female (n)	Female Mean ± SD	Female Median (Min.–Max.)	Male (n)	Male Mean ± SD	Male Median (Min.–Max.)	*p*
**0–216**	60	46.3 ± 57.8	25 (0–214)	60	48.6 ± 66.9	24 (0–216)	0.688 *
***Age subgroups (months)***							
**≥0–<1**	15	0.0 ± 0.0	0 (0–0)	15	0.0 ± 0.0	0 (0–0)	1.000 **
**≥1–<24**	15	10.5 ± 6.7	8 (1–22)	15	6.1 ± 5.3	4 (1–21)	0.039 **
**≥24–<60**	15	45.1 ± 8.3	44 (28–59)	15	38.1 ± 9.4	37 (27–59)	0.034 **
**≥60–<216**	15	129.5 ± 53.7	108(62–214)	15	150.4 ± 55.2	163(63–216)	0.309 **

* Student *t*-test, ** Mann–Whitney U test. The distribution of the 120 patients included in the study, by gender and age, is presented, along with mean ages and standard deviations, median and minimum and maximum values, and *p*-values by age group.

**Table 2:** Comparison of mean, SD, median, and *p*-values of venous and capillary acylcarnitine and amino acid levels in 120 patients included in the study.

Parameter	Venous (µmol/L) (Mean ± SD, Median, Min.–Max.)	Capillary (µmol/L) (Mean ± SD, Median, Min.–Max.)	*p* Value
**C0**	23.97 ± 12.19, 22.16 (5.90–111.29)	26.09 ± 11.82, 23.59 (6.96–84.60)	**0.003**
**C2**	15.17 ± 7.69, 13.40 (5.55–46.10)	16.37 ± 8.12, 14.29 (5.74–54.70)	**0.002**
**C3**	2.24 ± 5.26, 1.14 (0.37–42.24)	2.32 ± 5.23, 1.23 (0.28–39.88)	0.165
**C4**	0.20 ± 0.12, 0.16 (0.05–0.69)	0.21 ± 0.12, 0.18 (0.02–1.06)	0.274
**C5**	0.23 ± 0.99, 0.11 (0.03–10.89)	0.23 ± 0.69, 0.15 (0.05–7.68)	0.997
**C5.1**	0.02 ± 0.03, 0.01 (0.00–0.21)	0.02 ± 0.03, 0.01 (0.00–0.23)	0.994
**C5-DC**	0.17 ± 0.70, 0.06 (0.01–6.14)	0.15 ± 0.57, 0.06 (0.01–4.64)	0.271
**C5-OH**	0.11 ± 0.07, 0.09 (0.03–0.45)	0.10 ± 0.06, 0.09 (0.01–0.40)	0.182
**C6**	0.09 ± 0.38, 0.05 (0.00–4.06)	0.09 ± 0.42, 0.04 (0.01–4.59)	0.760
**C8**	0.04 ± 0.03, 0.03 (0.00–0.20)	0.03 ± 0.03, 0.03 (0.01–0.19)	**0.002**
**C8.1**	0.04 ± 0.03, 0.03 (0.00–0.19)	0.03 ± 0.03, 0.03 (0.01–0.19)	**0.001**
**C10**	0.07 ± 0.05, 0.06 (0.01–0.24)	0.06 ± 0.05, 0.06 (0.01–0.36)	**0.025**
**C10.1**	0.09 ± 0.06, 0.07 (0.01–0.54)	0.07 ± 0.04, 0.06 (0.01–0.23)	**0.001**
**C12**	0.07 ± 0.03, 0.06 (0.01–0.18)	0.06 ± 0.04, 0.06 (0.01–0.22)	0.054
**C14**	0.10 ± 0.06, 0.05 (0.01–0.33)	0.10 ± 0.07, 0.08 (0.01–0.41)	0.737
**C14.1**	0.07 ± 0.04, 0.06 (0.01–0.28)	0.06 ± 0.04, 0.05 (0.01–0.30)	**0.034**
**C14.2**	0.04 ± 0.02, 0.03 (0.00–0.15)	0.04 ± 0.02, 0.03 (0.01–0.17)	0.461
**C16**	0.88 ± 0.62, 0.69 (0.12–4.97)	0.97 ± 0.73, 0.78 (0.01–4.46)	**0.013**
**C16.1**	0.05 ± 0.04, 0.04 (0.01–0.22)	0.05 ± 0.07, 0.03 (0.00–0.51)	0.177
**C18**	0.54 ± 0.27, 0.51 (0.14–2.03)	0.57 ± 0.28, 0.54 (0.12–1.93)	0.064
**C18.1**	1.13 ± 0.57, 0.98 (0.32–3.63)	1.22 ± 0.66, 1.04 (0.10–4.58)	**0.026**
**C18.2**	0.46 ± 0.30, 0.42 (0.07–2.76)	0.48 ± 0.25, 0.44 (0.06–1.86)	0.235
**Arginine**	28.19 ± 23.69, 22.21 (4.59–147.13)	36.21 ± 22.11, 30.21 (12.03–189.80)	**<0.001**
**Aspartic acid**	84.49 ± 55.25, 70.34 (24.45–396.27)	55.86 ± 25.32, 51.30 (17.00–153.19)	**<0.001**
**Alanine**	180.10 ± 58.28, 169.22 (71.15–383.10)	183.70 ± 71.44, 166.66 (76.97–407.68)	0.534
**Citrulline**	27.45 ± 13.43, 24.71 (5.80–106.32)	34.29 ± 17.97, 31.87 (10.30–146.70)	**<0.001**
**Glutamic acid**	163.17 ± 60.34, 147.34 (71.16–343.35)	221.59 ± 79.35, 198.50 (104.00–468.00)	**<0.001**
**Glycine**	184.44 ± 71.13, 168.49 (89.61–471.66)	208.11 ± 89.13, 190.31 (10.50–462.00)	**0.002**
**Leu + Ileu**	94.76 ± 41.14, 84.54 (33.22–327.26)	99.22 ± 39.50, 92.32 (33.25–282.19)	**0.027**
**Methionine**	21.64 ± 54.17, 13.68 (5.60–487.60)	24.03 ± 57.66, 15.66 (5.75–483.84)	**0.028**
**Phenylalanine**	63.47 ± 116.57, 30.90 (4.64–743.32)	68.63 ± 120.10, 35.20 (10.03–841.16)	0.071
**Tyrosine**	82.13 ± 62.61, 64.62 (26.42–456.91)	92.33 ± 67.78, 77.69 (25.10–574.21)	**<0.001**
**Valine**	66.56 ± 35.45, 61.02 (22.80–343.29)	69.44 ± 28.23, 64.35 (25.86–252.21)	0.080
**Phe/tyr**	1.05 ± 2.54, 0.48 (0.01–22.26)	1.03 ± 2.44, 0.49 (0.02–18.51)	0.721
**Leu/phe**	3.30 ± 6.32, 2.64 (0.09–70.45)	2.70 ± 2.59, 2.55 (0.06–28.11)	0.093
**Met/phe**	0.83 ± 2.61, 0.44 (0.01–21.77)	0.74 ± 2.25, 0.43 (0.01–19.50)	**0.037**
**ASA**	0.02 ± 0.12, 0.00 (0.00–1.28)	0.04 ± 0.27, 0.00 (0.00–2.95)	0.393

Acylcarnitine and amino acid parameters, obtained simultaneously via venous and capillary sampling, were compared in 120 patients. Statistical analysis was performed using the Wilcoxon signed-rank test. Data with a *p*-value < 0.05 are indicated in bold. Phe; phenylalanine, Leu; leucine, Ileu; isoleucine, Tyr; tyrosine, Met; methionine, ASA; argininosuccinic acid.

**Table 3:** Comparison of disease-specific parameter measurements in patients diagnosed with inherited metabolic diseases, where pathological findings are expected in tandem mass spectrometry, in acylcarnitine and amino acid analyses obtained via venous and capillary methods.

Parameter	Specific Inherited Metabolic Disease (Mean ± SD, Median, Min.–Max.) (µmol/L)	*p*-Value (Venous vs. Capillary)
**Phenylketonuria or Hyperphenylalaninemia Patients (n = 17)**		
Phe (Venous)	244.13 ± 240.82/160.03 (4.64–743.32)	0.431
Phe (Capillary)	261.46 ± 245.57/187.65 (10.03–841.16)	
Phe/Tyr (Venous)	4.42 ± 5.79/2.98 (0.01–22.26)	0.517
Phe/Tyr (Capillary)	4.43 ± 5.47/2.77 (0.02–18.51)	
**Methylmalonic acidemia patients (n = 6)**		
C3 (Venous)	21.25 ± 13.83/22.81 (5.42–42.24)	0.688
C3 (Capillary)	21.84 ± 12.66/25.23 (6.65–39.88)	
**Glutaric aciduria type 1 patients (n = 3)**		
C5DC (Venous)	3.92 ± 2.67/4.67 (0.95–6.14)	0.374
C5DC (Capillary)	3.31 ± 1.93/4.20 (1.10–4.64)	
**Argininemia patients (n = 1)**		
Arginine (Venous)	110.83 ± 0.00/110.83 (110.83–110.83)	N/A *
Arginine (Capillary)	189.80 ± 0.00/189.80 (189.80–189.80)	
**Argininosuccinate lyase deficiency patient (n = 1)**		
ASA (Venous)	1.28 ± 0.00/1.28 (1.28–1.28)	N/A *
ASA (Capillary)	2.95 ± 0.00/2.95 (2.95–2.95)	
Citrulline (Venous)	106.32 ± 0.00/106.32 (106.32–106.32)	N/A *
Citrulline (Capillary)	146.70 ± 0.00/146.70 (146.70–146.70)	
**Hereditary tyrosinemia type 1 patients (n = 2)**		
Tyrosine (Venous)	380.42 ± 95.45/380.42 (312.92–447.91)	0.186
Tyrosine (Capillary)	456.10 ± 167.03/456.10 (337.99–574.21)	
**Homocystinuria patients (n = 2)**		
Methionine (Venous)	429.83 ± 81.69/429.83 (372.06–487.60)	0.655
Methionine (Capillary)	461.92 ± 31.00/461.92 (440.00–483.84)	
**Isovaleric acidemia patient (n = 1)**		
C5 (Venous)	10.89 ± 0.00/10.89 (10.89–10.89)	N/A *
C5 (Capillary)	7.68 ± 0.00/7.68 (7.68–7.68)	

In tandem mass spectrometry analysis, the mean ± SD, median, min.-max., and *p*-value analyses of disease-specific acylcarnitine and amino acid levels measured in venous and capillary blood of patients with disease-specific diagnostic parameters and definitive diagnosis were performed using the Wilcoxon signed-rank test and are summarized in the table above. Phe: phenylalanine, Tyr: tyrosine, ASA: argininosuccinic acid, N/A * (Not Applicable): means that could not be evaluated as there was only one patient in the relevant group, min.: minimum, max.: maximum.

**Table 4:** Comparison of statistically significant parameters according to age subgroups and venous/capillary sample collection method.

Parameters in Which Statistically Significant Differences Were Found	Venous	Capillary	*p*-Value
**Age; ≥0–<1 Month**			
C0; C2; C4; C5; C16; C18; C18:1	**↓**	**↑**	*p* = 0.022; *p* = 0.003; *p* = 0.014; *p* < 0.001; *p* = 0.008; *p* = 0.031; *p* = 0.013
Arg; Phe; Tyr; Val			*p* = 0.001; *p* = 0.023; *p* = 0.002; *p* = 0.001
C8.1; Asp; Leu/phe	**↑**	**↓**	*p* = 0.004; *p* = 0.006; *p* = 0.007
**Age; ≥1–<24 Months**			
C0; C5	**↓**	**↑**	*p* = 0.038; *p* = 0.009
Arg; Cit; Glu; Gly; Leu + Ile; Met; Phe; Tyr; ASA			*p* = 0.001; *p* = 0.001; *p* = 0.001; *p* = 0.038; *p* = 0.041; *p* = 0.003; *p* = 0.001; *p* = 0.001; *p* = 0.042
C8	**↑**	**↓**	*p* = 0.007
**Age; ≥24–<60 Months**			
C0; C5; C10; C14	**↓**	**↑**	*p* = 0.028; *p* = 0.026; *p* = 0.029; *p* = 0.016
Arg; Cit; Glu; Phe			*p* = 0.001; *p* = 0.001; *p* = 0.001; *p* = 0.033
Leu/phe	**↑**	**↓**	*p* = 0.009
**Age; ≥60–<216 Months**			
Arg; Cit; Glu; Met; Phe; Tyr	**↓**	**↑**	*p* = 0.013; *p* = 0.007; *p* < 0.001; *p* = 0.029; *p* = 0.022; *p* = 0.008
C8; C8.1; C10; C10.1; C12; C14; C14.1	**↑**	**↓**	*p* = 0.004; *p* = 0.013; *p* = 0.005; *p* = 0.001; *p* < 0.001; *p* = 0.005; *p* = 0.001
Asp; Leu/phe			*p* = 0.016; *p* = 0.005

In age-stratified subgroups with equal numbers of patients of each gender, parameters studied using venous and capillary methods that showed statistically significant differences were summarized by low and high values, along with *p*-values. Statistical analysis was performed using the Wilcoxon signed-rank test. Arg: Arginine, Cit: Citrulline, Ile: Isoleucine, Leu: Leucine, Val: Valine, Phe: phenylalanine, Tyr: Tyrosine, Asp: Aspartic acid, Glutamic acid: Glu, ASA: Argininosuccinic acid, Methionine: Met.

**Table 5:** Venous–Capillary Difference Direction and Consistency Analysis by Age Groups.

Parameter	0–1 Month (n = 30)	1–24 Month (n = 30)	24–60 Month (n = 30)	60–216 Month (n = 30)	Consistency
C0	Cp > V *	Cp > V *	Cp > V *	Cp > V	consistent
**C2**	Cp > V *	Cp > V	Cp > V	V > Cp	**inconsistent**
**C3**	Cp > V	Cp > V	Cp > V	V > Cp	**inconsistent**
C4	Cp > V *	Cp > V	Cp > V	Cp > V	consistent
C5	Cp > V *	Cp > V *	Cp > V *	Cp > V	consistent
**C8**	Cp > V	V > Cp *	V > Cp	V > Cp *	**inconsistent**
**C8.1**	V > Cp *	V > Cp	Cp > V	V > Cp *	**inconsistent**
C10	V > Cp	V > Cp	V > Cp *	V > Cp *	consistent
C10.1	V > Cp	V > Cp	V > Cp	V > Cp *	consistent
**C12**	Cp > V	V > Cp	V > Cp	V > Cp *	**inconsistent**
**C14**	Cp > V	Cp > V	V > Cp *	V > Cp *	**inconsistent**
**C16**	Cp > V *	Cp > V	Cp > V	V > Cp	**inconsistent**
**C18**	Cp > V *	Cp > V	Cp > V	=	**inconsistent**
C18:1	Cp > V *	Cp > V	Cp > V	Cp > V	consistent
Arginine	Cp > V *	Cp > V *	Cp > V *	Cp > V *	consistent
Aspartic acid	V > Cp *	V > Cp *	V > Cp *	V > Cp *	consistent
Glutamic acid	Cp > V *	Cp > V *	Cp > V *	Cp > V *	consistent
Citrulline	Cp > V *	Cp > V *	Cp > V *	Cp > V *	consistent
Tyrosine	Cp > V *	Cp > V *	Cp > V	Cp > V *	consistent
Phenylalanine	Cp > V *	Cp > V *	Cp > V *	Cp > V *	consistent
Leu/Phe	V > Cp *	V > Cp *	V > Cp *	V > Cp *	consistent

Cp; capillary, V; venous, Cp > V: Capillary is higher; V > Cp: Venous is higher; *: Wilcoxon *p* < 0.05. Parameters with directional inconsistency are shown in bold.

## Data Availability

The original contributions presented in this study are included in the article. Further inquiries can be directed to the corresponding authors.

## Abbreviations

The following abbreviations are used in this manuscript:

MS/MS	Tandem mass spectrometry
IV	Intravenous
TAP	Touch-Activated Phlebotomy
PKU	Phenylketonuria
HPA	Hyperphenylalaninemia
IVA	Isovaleric academia
MMA	Methylmalonic acidemia
ASL	Argininosuccinate lyase
Asp	Aspartic Acid
Arg	Arginine
Ala	Alanine
Cit	Citrulline
Glu	Glutamic Acid
Gly	Glycine
Leu	Leucine
Ileu	Isoleucine
Met	Methionine
Phe	Phenylalanine
Tyr	Tyrosine
Val	Valine
